# Animated VR and 360-degree VR to assess and train team sports decision-making: a scoping review

**DOI:** 10.3389/fpsyg.2024.1410132

**Published:** 2024-07-15

**Authors:** Yaxiang Jia, Xuan Zhou, Jing Yang, Quan Fu

**Affiliations:** Capital University of Physical Education and Sports, Beijing, China

**Keywords:** decision-making, virtual reality, review, team sport, sport decision

## Abstract

**Introduction:**

In team sports, athletes’ ability to make quick decisions plays a crucial role. Decision-making proficiency relies on the intricate balance of athletes’ perceptual and cognitive abilities, enabling them to assess the competitive environment swiftly and select the most appropriate actions from various options. Virtual reality (VR) technology is emerging as a valuable tool for evaluating and refining athletes’ decision-making skills. This study systematically examined the integration of VR technology into decision-making processes in team sports, aiming to identify more effective methods for presenting and interacting with virtual decision-making systems, thus enhancing the evaluation and refinement of athletes’ decision making abilities.

**Methods:**

Following the preferred reporting items for systematic reviews and meta-analyses (PRISMA) guidelines, a thorough search of respected research databases, including Web of Science, PubMed, SPORTDiscus, ScienceDirect, PsycINFO, and IEEE, was conducted using carefully selected keywords.

**Results:**

Twenty research papers meeting predefined inclusion criteria were included after careful evaluation. These papers were systematically analyzed to delineate the attributes of virtual decision-making task environments, the interactive dynamics inherent in motor decision-making tasks, and the significant findings

**Discussion:**

This review indicate that (1) the effectiveness of VR technology in assessing and improving athletes’ decision-making skills in team sports; (2) the construction of virtual environments using the Head-Mounted Display (HMD) system, characterized by enhanced ease and efficiency; (3) the potential for future investigations to explore computer simulations to create more expansive virtual motion scenarios, thus efficiently generating substantial task scenario material, diverging from the constraints posed by 360-degree panoramic videos; and (4) the integration of motion capture technology for identifying and monitoring athletes’ decision-making behaviors, which not only enhances ecological validity but also augments the transfer validity of virtual sports decision-making systems. Future research endeavors could explore integrating eye-tracking technology with virtual reality to gain insights into the intrinsic cognitive-action associations exhibited by athletes.

## 1 Introduction

Decision-making is a crucial mental skill necessary for peak athletic performance. It involves understanding and interpreting game-related information accurately, which helps in making the right choices for sport-specific actions (Baker et al., 2003). In addition, a vital ability for excelling as a player in team sports is to effectively analyze defensive strategies, which requires understanding the opponent’s intentions and responding quickly and accurately (Hohmann et al., 2016). Scientific studies show that training aimed at improving perception and cognition can enhance athletes’ decision-making abilities in competitive sports. The Modified Perceptual Training Framework (MPTF) (Hadlow et al., 2018) is a theoretical model that guides the development of targeted training programs by identifying important factors related to visual and perceptual training. The MPTF consists of three main components: “Targeted Perceptual Function,” “Stimulus Correspondence,” and “Response Correspondence,” which form the basis of visual and perceptual training methods (Discombe et al., 2022). (1) “Targeted perceptual function” refers to specific perceptual skills that need improvement through training. Current research suggests that training focused on higher-order perceptual skills, like sport-specific prediction, may be more effective than training on general visual skills such as acuity. (2) ‘Stimulus correspondence’ refers to the similarity between stimuli used in training and those encountered in real game situations. Therefore, designing training tasks that accurately reflect real-world conditions is crucial for optimizing training effectiveness. (3) Similarly, ‘response correspondence’ describes how closely the responses required in training match those needed in actual athletic activities. Unlike verbal or written responses, authentic motor actions are essential for connecting perception to action, thus improving the accuracy of perceptual-cognitive performance (Hadlow et al., 2018).

The most effective way to improve athletes’ decision-making skills involves using simulation training directly on the training field, where athletes engage in self-directed exploration of decision-making processes (Neumann et al., 2018). However, this approach has significant drawbacks, including being time-consuming and labor-intensive, and the decision-making scenarios may lack repeatability and controllability. To overcome these limitations, video-based training has become a popular strategy for enhancing decision-making skills. This method uses video presentations to replicate game scenarios, allowing athletes to practice without physically performing the skills (Zhao et al., 2022). However, a major challenge with video-based training is that the videos are often captured from a distant third-person viewpoint, which reduces the fidelity of decision-making tasks compared to real-game scenarios (Pinder et al., 2011). The main goal of video-based decision-making tasks is to help athletes make quick decisions in a context that closely resembles real games. To improve the representativeness of video-based training tasks, researchers need to address these limitations and enhance the ecological validity of their research instruments (Putranto et al., 2023).

Scientific evidence backs the effectiveness of virtual reality (VR) as a video-based training method. Athletes who train in interactive virtual environments show better performance compared to those using traditional video images (Vignais et al., 2015). Virtual Reality (VR) is defined as a computer-generated simulation of a three-dimensional environment that allows users to interact with the virtual space through sensory stimuli and devices such as head-mounted displays, motion controllers, and haptic feedback systems (Faure et al., 2020). This technology aims to create an immersive experience by providing real-time responses to user actions, thus fostering a sense of presence within the artificial environment (Le Noury et al., 2021). Head-Mounted Displays (HMDs) and CAVE systems (Cave Automatic Virtual Environment) are widely used virtual environments in the field of sports (Slater, 2009). A Head-Mounted Display (HMD) is a wearable gadget that projects virtual environments directly in front of the eyes through a head-worn device like a VR headset. This device shields the eyes, eliminating external visual distractions, and incorporates one or more small screens to provide a stereoscopic view of the virtual world with a wide-angle perspective. This method, representing the most common approach to experiencing virtual reality, offers users a high level of immersion through integrated screens, lenses, and sensors (Ghinea et al., 2018). By combining HMD with head tracking, users can observe all aspects of the virtual environment, achieving 360-degree visual tracking by rotating their head (Putranto et al., 2023). The CAVE system, consisting of multiple large projection screens, projects computer-generated 3D graphics onto the walls and floor of a room, creating an enclosed virtual environment (Steuer, 1992).

In prior research, the classification of VR has been delineated into Animated VR and 360-Degree VR, based on the nature of the virtual content (Le Noury et al., 2021). There are noteworthy distinctions between them. Animated VR involves computer-generated environments where scenes dynamically adapt in response to user interactions with virtual entities. Conversely, 360-Degree VR consists of pre-recorded video segments capturing real-world environments from a static perspective, allowing users only to view these segments without interaction capabilities (Le Noury et al., 2021). Therefore, the ability of virtual reality technology to create realistic and repeatable sports decision-making scenarios makes it a powerful tool for evaluating and refining sports decision-making skills. It is widely used in team training programs (Yunchao et al., 2023). Nonetheless, due to variations in decision-making tasks, research subjects, and VR technologies across studies, the intervention effects have not clearly synthesized. Hence, this study conducts a systematic review of VR’s application in team sports decision-making. It investigates how to select the most suitable VR technologies to better represent virtual decision-making tasks and appropriate interaction methods, aiming to integrate additional technologies to further improve the ecological validity of research methodologies and profoundly examine the intrinsic links between athletes’ cognition and action.

## 2 Methods

### 2.1 Study design

The study rigorously adhered to the PRISMA guidelines for systematically evaluating pertinent experimental studies. Research inquiries were organized using the Population, Intervention, Comparison, and Outcome (PICO) tool. Population consisted of athletes or referees; Intervention involved the use of Animated VR and 360-Degree VR as assessment and training tools; Comparison entailed the use of images or standard 2D videos as assessment and training decision-making tools; Outcome focused on the accuracy and speed of decision-making.

This systematic review aims to explore the effectiveness of virtual reality technology in evaluating and enhancing athletes’ decision-making skills. Moreover, our objective is to pinpoint more effective methods for presenting and engaging with virtual decision-making scenarios. Lastly, we will scrutinize the constraints of virtual reality technology in team sports decision-making and suggest avenues for future research.

### 2.2 Search methods

We systematically searched through prominent English databases—Web of Science, PubMed, SPORTDiscus, Sincedirect, PsycINFO, and IEEE–to gather studies published until January 31, 2024. For all mentioned databases, the search was conducted using different combinations of the following search terms:[(“virtual reality” OR “Reality, Virtual “OR “Virtual environment” OR “Virtual reality technology” OR “Virtual world” OR “Virtual systems” OR “Virtual appliance” OR “Immersive environments” OR “Immersive devices” OR “Animated Virtual reality” OR “360-Degree Virtual reality “OR “Reality, Instructional Virtual” OR “Virtual Realities, Instructional”) AND (“Sport Decision-making” OR “Decision-making Training” OR “sports decision “OR “decision training “OR “team decision “OR “decision-making “)].

### 2.3 Eligibility criteria

Two authors collaborated to establish the inclusion and exclusion criteria, followed by a thorough independent review and evaluation of the studies. The selection process adhered to stringent inclusion criteria: (1) Articles had to be in English; (2) Publications needed to be research or journal articles; (3) Only full-text published articles were considered; (4) Studies had to involve a group of healthy competitive athletes or referees; (5) At least one type of motor decision-making skill must be included and required clear metrics for measuring decision-making performance(e.g., the timing of passing, dribbling and shooting in basketball); and (6) The study must incorporate a VR technology intervention. Exclusion criteria included: (1) Absence of experimental methods and findings; (2) Insufficient details on the VR environment or technology in articles (e.g., formation of virtual environments not reported); and (3) Studies not directly related to the specific domain of team sport decision-making.

### 2.4 Selection process

All identified literature was exported into Endnote20. Following the removal of duplicates, two researchers independently examined the titles and abstracts of the studies for further screening, excluding those not meeting the study criteria. The remaining literature underwent a comprehensive full-text assessment to determine its alignment with the research criteria. In cases where discrepancies arose between the two researchers regarding inclusion in the assessment process, consultation with a third researcher was sought to mitigate bias. Two researchers independently conducted data extraction for each study, and any differences were discussed and resolved through consultation with the third researcher.

### 2.5 Extraction of data

The integrated studies were independently analyzed by the two authors to extract the following details: (1) Researcher information (including year and country); (2) Participant characteristics (age and exercise level); (3) Exercise program details; (4) Type of VR utilized (including virtual environment (VE) creation and interaction devices); (5) Nature of team sport decision-making content (decision-making tasks and forms of interactions); (6) Implementation of additional artificial intelligence (AI) devices; and (7) Principal findings.

## 3 Results

### 3.1 Study selection

**Figure 1:**
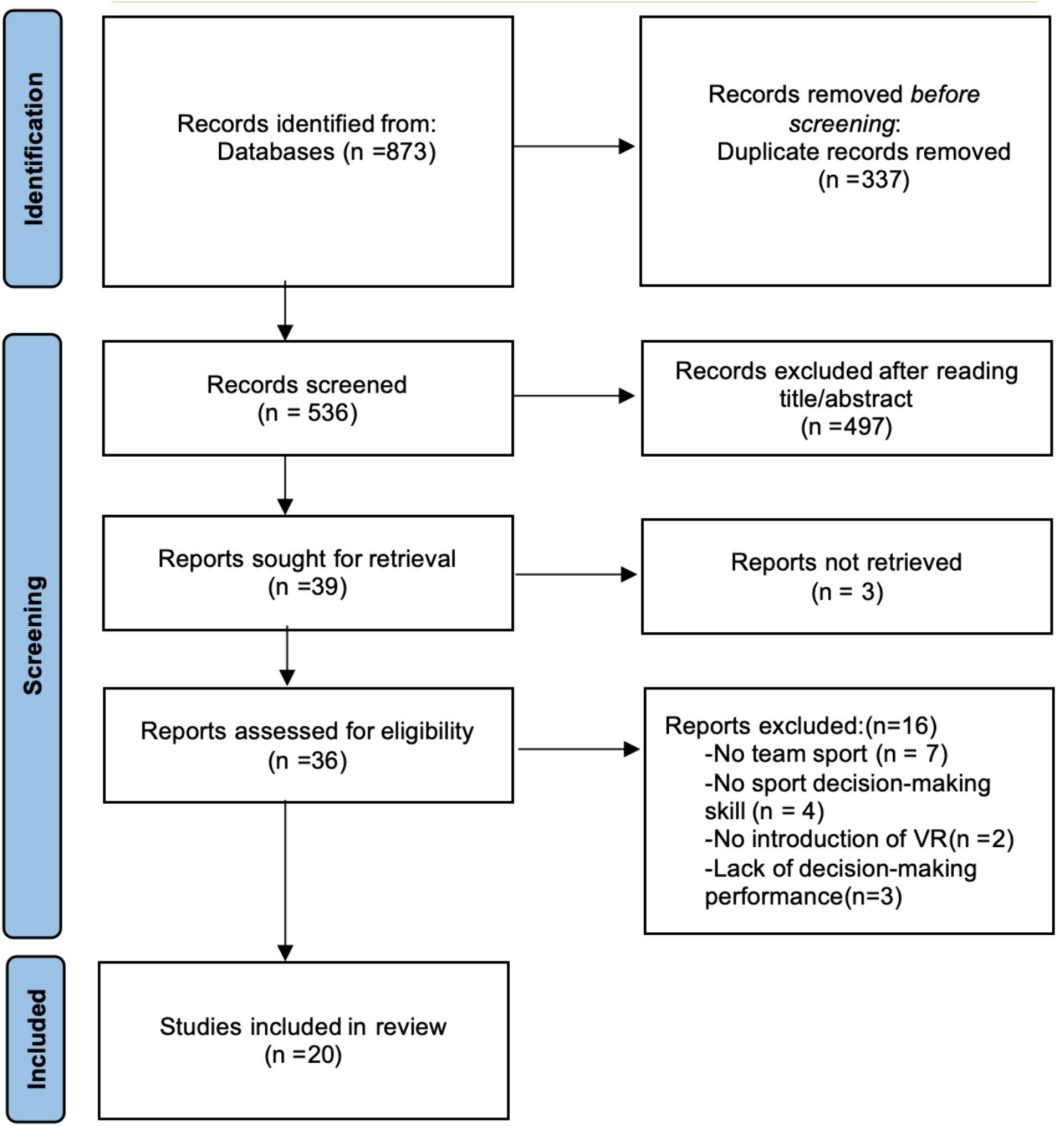
Flow chart illustrating the following steps of the study selection.

A total of 873 papers were found initially in English electronic databases. After removing duplicate literature, 536 research papers were examined based on title and abstract. Among these, 39 papers met the criteria for moving to the next stage. The two authors conducted a full-text search of these 39 documents, excluding three that could not be fully accessed. Subsequently, 36 pieces of literature were independently evaluated using predetermined inclusion and exclusion criteria, resulting in the exclusion of 16. The remaining 20 were considered suitable for inclusion in this study. Following the PRISMA flowchart, the authors chose these 20 papers as samples for further discussion to support this study. This systematic literature review was based on these 20 research papers for academic reference and learning. Figure 1 outlines the retrieval and screening process.

### 3.2 Characteristics of included studies

This systematic review encompasses 20 studies, with a predominant focus on the past decade. The total number of participants across these studies amounts to 570 individuals. The team sports examined include football, cricket, basketball, baseball, handball, and rugby.

Table 1 summarizes the characteristics of participants in various studies. These investigations often applied rigorous criteria, primarily focusing on athletes or referees with specific motor skill proficiency. The selection of participants with comparable skill levels was guided by the research objectives and the unique attributes of each sport. For example, Marshall et al. (2023) investigated the enhancement of soccer players’ heading decision-making skills through virtual reality (VR) training. They divided soccer players with similar levels of ability into a VR training group and a no-training group, enabling an assessment of the impact of VR training (Marshall et al., 2023). The expert-novice paradigm is a common approach in selected studies. For instance, Kittel et al. (2019) sought to identify the most suitable decision-making assessment tool for soccer referees. The study involved 13 elite-level soccer referees and 15 amateur referees, participating in a decision-making task within two experimental settings while comparing virtual reality 360-degree video and match broadcast video (Kittel et al., 2019).

**Table 1:** Table of methodological characteristics and main results of studies included in the analysis.

Study	Country	Sports	Experience level	Gender (Male/Female)	*N*	VE (interactive device)	Other devices	Decision-making task	Outcome measure	Conclusions
Marshall et al. (2023)	United Kingdom	Soccer	Amateur players	Control(16/2) VR(14/4)	38	Aminated VR in HMD	No	Behavioral interaction responses to header shot decisions	Goal scoring, shooting accuracy and self-efficacy	VR header training enables players to enhance their heading abilities without experiencing repetitive head impacts.
Van Biemen et al. (2023)	Netherlands	Soccer	Sub-elite referees	15/0	15	Aminated VR in HMD	Eye-tracking device	Oral decision-making options	Decision accuracy, visual behavior	Compared to video footage, VR comes closer to reproducing the real environment, making it an effective tool for football referee training.
Theofilou et al. (2022)	Greece	Soccer	Collegiate level players	38/0	38	Aminated VR in HMD	Illuminated sensor	Perform the appropriate action	Response time	Improved attention, concentration, and physical fitness can enhance reaction time (RT), leading to better athletic performance. The VR technique can be used for a quick assessment of RT.
Discombe et al. (2022)	United Kingdom	Cricket	Amateur	14/4	18	360‑Degree VR in HMD	No	Verbal prediction of ball landing location	Prediction accuracy and confidence	Immersive video can effectively assess and train cricket prediction and decision-making skills.
Tsai et al. (2021)	China-Taiwan	Basketball	Amateur players	36/9	45	Animated VR and 360‑Degree VR in HMD	Inertial Measurement Unit	Action responses for decision-making	Decision accuracy, decision time	To save time and effort, it is recommended to use animated scenarios created from computer simulations instead of 360^o^ videos to train motor skills.
Fortes et al. (2021)	Brazil	Soccer	National level	26/0	26	360‑Degree VR in HMD	Eye-tracking device	Action responses for decision-making	Decision accuracy, visual search behavior	VR demonstrates greater improvement in decision-making and visual search behavior compared to VID.
Hosp et al. (2021)	Germany	Soccer	Expert, advanced, and novice players	Not reported	33	360‑Degree VR in HMD	Eye-tracking device	Verbal reporting of decision-making responses	Decision accuracy	By analyzing the eye movement characteristics of goalkeepers during virtual reality decision-making tests, it is possible to differentiate effectively between athletes of different levels.
Kittel et al. (2021)	Australia	Australian rules football	Elite football umpires	21/0	21	360‑Degree VR in HMD	No	Verbal reporting of decision-making responses	Decision accuracy, rate of unexplained decisions	Neither of the video-based tests has the sensitivity to distinguish between elite referees.
Kittel et al. (2020)	Australia	Australian rules football	Elite football umpires	32/0	32	360‑Degree VR in HMD	No	Verbal reporting of decision-making responses	360 VR test performance; Match broadcast test performance	Neither the VR group nor the traditional video group showed significant improvement. However, the VR group scored higher than the control group on the realism test, indicating that VR could be a suitable training tool.
Magnaguagno and Hossner (2020)	Switzerland	Handball	Expert, near expert	24/0	24	360‑Degree VR in CAVE	Motion Capture System	Selecting defensive decisions through body movement	The detection of patterns, the correctness of motor responses	Experts are more capable of evaluating the quality of their teammates’ defenses and adjusting based on past experiences, while those who are not quite specialists rely more heavily on the quality of their teammates’ defenses.
Kittel et al. (2019)	Australia	Australian rules football	Elite and amateur football umpires	28/0	28	360‑Degree VR in HMD	No	Verbal reporting of decision-making responses	Decision accuracy	VR is a more ecologically valid method for assessing the decision-making process during games.
Pagé et al. (2019)	Canada	Basketball	Elite basketball players	21/6	27	360‑Degree VR in HMD	No	Answer question verbally	Decision accuracy	2D video training decisions cannot be generalized to real scenarios, while VR training decisions can be both migrated to real scenarios and yield generalization benefits.
Panchuk et al. (2018)	Australia	Basketball	Elite basketball players	9/9	18	360‑Degree VR in HMD	No	Verbalize their decision and were given a ball to simulate their decision	Immersive test performance; small side game performance	The study found that the immersive video training group did not demonstrate significant improvement. However, the use of immersive video as a perceptual training tool is recommended, and further exploration of its differences from other training modalities is needed.
Wirth et al. (2018)	Germany	Soccer	Low-skilled group and high-skilled soccer players	30/0	30	360‑Degree VR in HMD	No	The verbal decision	Decision response time, accuracy	High-level athletes made decisions significantly faster than low-level athletes, with no significant difference in decision accuracy. The decision-making process was more popular among the athletes.
Isogawa et al. (2018)	Germany	Baseball	High level baseball player	3/0	3	360o VR and animated VR in HMD	Xsens MVN	Actions to make a hitting decision	Decision response time, accuracy	360VR and animated VR have the potential to replace real-life motion environments.
Van Maarseveen et al. (2018)	Netherlands	Soccer	Highly talented soccer players	0/22	22	360‑Degree VR in CAVE	Eye-tracking device	Select the button that represents the corresponding action	Visual gaze behavior, decision performance	The scores of perceived-cognitive skills tests are not indicative of the on-field performance of soccer players.
Gray (2017)	America	Baseball	Collegiate level players	80/0	80	360‑Degree VR in CAVE	Position tracker sensor	Decisions based on the angle and distance of the incoming ball.	Batting Performance Test	Training in virtual reality environments can improve athletic performance.
Hohmann et al. (2016)	Germany	Handball	National level	30/20	50	360‑Degree VR in CAVE	No	The verbal decision	Decision response time, accuracy	3D video training is a useful tool for improving handball selection time, but it does not necessarily improve decision quality. This is because stimulus form plays a crucial role in rapid selection.
Vignais et al. (2015)	France	Handball	Elite level	10/0	10	Aminated VR in CAVE	Motion Capture System	Recording of subjects’ ball-blocking decisions by a motion-capture system	Decision response time, accuracy	Virtual reality research instruments are more effective than video-based instruments in analyzing the visual information acquisition of handball goalkeepers.
Watson et al. (2011)	United Kingdom	Rugby	Novice	8/6	14	Aminated VR in HMD	Motion Capture System	Selecting decision-making moments by pressing buttons	Accuracy of judgment	Expert decision makers can assess the feasibility of an action using optical variables and make prompt decisions with high accuracy.

Among the eight studies involving female subjects, only two conducted a comparative analysis of decision-making performance across genders in different settings. Panchuk et al. (2018) examined the effectiveness of immersive video training and compared its effects between male and female athletes. Hohmann et al. (2016) investigated the efficacy of 3D video tools for training decision-making skills outside controlled environments, dividing 30 male and 20 female handball players into three groups: 3D video, 2D video, and traditional tactical board. The study aimed to determine the optimal training method for decision-making skills.

Overall, the comprehensive analysis of these studies reveals a growing emphasis on advanced training methods such as virtual reality and immersive video, which are gaining traction for their potential to significantly enhance decision-making skills in sports. The inclusion of both elite and amateur participants, as well as the consideration of gender differences, highlights a commitment to understanding the diverse factors that influence athletic performance. By continuously exploring and validating new approaches, researchers and practitioners can better support the development of decision-making skills, ultimately enhancing performance in various team sports.

### 3.3 Construction of different virtual reality environments

Table 1 shows that 15 out of 20 studies utilized HMDs to create virtual reality environments. Among these, 11 studies employed a presentation method that captured the actual motion scene in advance from the first viewpoint using a 360-degree panoramic video device. The recorded video was then presented to the user through the HMD. For instance, in a series of studies by Kittel et al., researchers utilized a 360-degree panoramic video viewed through an HMD to generate virtual environments. They utilized a stationary 360-degree camera mounted on a tripod about 1.5 meters above the ground for filming. To ensure optimal visual correspondence of the task, the camera was positioned similarly to a referee in a match, approximately 15 meters from the side of the activity (Kittel et al., 2019, 2020, 2021). Additionally, six studies employed computers to create virtual reality animated scenarios, such as VR games or programs, which were presented to users through HMDs. Van Biemen et al. (2023) investigated the visual behavioral differences in soccer referees’ decision-making between virtual reality and real scenarios. They created virtual reality environments by having referees make decisions in an HMD while participating in a first-person view VR soccer game. The study aimed to evaluate the effectiveness of virtual reality technology as a tool for soccer referees’ decision-making training. Some studies utilized motion capture technology to develop specialized VR programs, in addition to using pre-existing VR games (Watson et al., 2011). In Watson’s study, the virtual rugby environment was created in collaboration with Université Rennes 2 and Queen’s University Belfast. This environment featured two avatars positioned facing the participant on a regulation rugby pitch within a seated stadium complete with advertising boards. Motion capture technology was used to record the authentic movements of player avatars, controlling their speed, motion paths, trajectories, and appearance, before integrating them into the virtual rugby field (Watson et al., 2011).

Moreover, two studies investigated the coherence between the two methods used in constructing virtual environments. Isogawa et al. exposed participants to four different environmental stimuli: Real Environment, Flat 2D Videos, 360 Panoramic Videos, and Animated VR for Reproduction of Real Space. They assessed athletes’ decision-making in batting, timing control, and positioning to determine whether Animated VR or 360-Degree VR more effectively replicated the real environment (Isogawa et al., 2018). Tsai et al. evaluated the effectiveness of traditional tactical board training, VR360-degree panoramic video training, and computer-generated virtual animation training in enhancing the motor decision-making abilities of basketball players (Tsai et al., 2021).

In sports research, the CAVE projection systems an alternative to HMDs that are sometimes used. Among the 20 studies analyzed, only five employed the CAVE projection system for constructing virtual environments. Researchers displayed pre-recorded 360-degree panoramic videos through the CAVE system, immersing athletes in a closed, non-interactive projection environment. Magnaguagno et al. developed a customized CAVE system with six clustered workstations and 11 projectors (Barco F50, 2560×1600, 60 Hz) projecting onto the front wall (6.00 m × 3.75 m), two side walls (11.00 m × 3.75 m), and the floor (6.00 m × 11.00 m). Two groups of handball players at varying skill levels viewed pre-recorded 360-degree panoramic videos within an immersive virtual environment created by the CAVE system. Subsequently, they underwent defensive decision-making tests and assessed decision quality using a motion capture system (Magnaguagno and Hossner, 2020). Hohmann et al. used a mobile 3D cinema-like projection system to present videos, which included two projectors, wireless polarized glasses, and a high-performance laptop. The screen measured 1.80 m × 2.40 m, positioned 4 m from the athlete, enhancing the viewing angle and detail observation (Hohmann et al., 2016). Additionally, one study employed the VICON MX40 motion capture system (Oxford Metrics, Oxford, United Kingdom) to capture the kinematic data of real athletes, which was transferred to the MKM (Manageable Kinematic Motions) animation engine. This produced a virtual animated version of the athlete, displayed through a projection system consisting of an SGI 83 Onyx2 Infinite Reality and a semi-cylindrical screen (radius 3.80 m, height 2.38 m, field of view 135°) situated 4.50 m from the participants. Viewers experienced stereoscopic vision using active glasses that alternately occluded each eye at a 120 Hz synchronization (Vignais et al., 2015).

### 3.4 Experimental results on evaluating and training sports decision-making in different virtual environments

Table 1 illustrates the four environments utilized for motor decision-making training: viewing a 360-degree panoramic video via an HMD or CAVE system and observing a computer-generated animated scenario through an HMD or CAVE system.

Researchers conducted comparative studies on these training environments for sports decision-making. Some studies found that using the CAVE system effectively improves athletes’ decision-making speed, though it does not significantly enhance the quality of their decisions (Hohmann et al., 2016). Hohmann et al. (2016) conducted a study employing a CAVE projection system to develop a virtual reality environment tailored for handball decision-making training. Participants were divided into three groups: one experienced 3D video, another observed 2D video, and the third utilized traditional tactical boards. Results revealed that the 3D video group exhibited faster decision-making times than the 2D video group, with no significant difference in decision quality. Compared to traditional tactical board training, both groups undergoing video training were effective; however, the 3D video training group demonstrated quicker decision-making. This indicates that incorporating 3D video training is effective in enhancing the decision-making speed of handball players without compromising decision quality. It is noteworthy that the stimulus form in the scenario plays a crucial role in rapid decision-making (Hohmann et al., 2016). In subsequent studies, researchers utilized virtual training environments in HMDs, employing both 360VR videos and Animated VR. These findings were corroborated in further research (Wirth et al., 2018). Wirth et al. (2018) developed a virtual reality environment using 360-degree panoramic videos displayed in HMDs and assessed the decision-making time and quality of soccer players at different skill levels. Additionally, they examined six different virtual scenario interaction methods to determine the most effective approach for soccer assessment and training in terms of user experience, presence, and immersion. Their results indicated that high-level soccer players had significantly faster decision-making times compared to lower-level players, although there was no significant difference in decision accuracy. Tsai et al. designed immersive training environments for basketball decision-making training by varying the training environments and methods, including traditional tactical boards, 360VR, and Animated VR. The findings suggested that while there was no significant difference in the acquisition of decision-making knowledge between traditional and VR-based methods, improvement in decision-making speed was evident (Wirth et al., 2018).

Other studies have raised concerns regarding the use of 360VR videos to evaluate athletes’ perceptual-cognitive abilities, specifically questioning whether performance in these assessments can predict actual performance in real matches. Van Maarseveen et al. (2018) conducted perceptual-cognitive tests on soccer players and analyzed their gaze behavior to better understand if performance in these tasks correlates with underlying processes. The study concluded that predicting athletes’ real-match performance based solely on perceptual-cognitive test results is not straightforward. These tests may not be as indicative of actual performance as previously thought (Van Maarseveen et al., 2018).

Pagé et al.’s study revealed that the improved motor decision-making ability gained through VR video training can be applied not only to real-game scenarios but also yields generalized benefits. The results demonstrate the effectiveness of using video simulation to enhance athletes’ decision-making and validate that the incorporation of virtual reality technology can further enhance training benefits (Pagé et al., 2019). However, some studies have indicated that the impact of VR video training on enhancing athletes’ decision-making abilities may not be substantial. Panchuk et al. (2018) observed that basketball players’ decision-making performance in real matches showed no significant change before and after VR video training, though there were notable improvements during immersive testing sessions. This discrepancy could be attributed to multiple factors (e.g., study design), diverging from the original research hypothesis (Panchuk et al., 2018). Nonetheless, the researchers maintained that immersive video training did not adversely affect athletes. Recognizing its potential value, they proposed using immersive VR video training as a tool for perceptual-cognitive training (Panchuk et al., 2018). Other studies have concurred with this perspective (Vignais et al., 2015; Hohmann et al., 2016; Gray, 2017; Isogawa et al., 2018; Kittel et al., 2019; Fortes et al., 2021; Hosp et al., 2021; Discombe et al., 2022), considering immersive VR videos as an effective means for assessing and training sports decision-making.

### 3.5 Interaction methods in different research decision-making tasks

Out of the 20 analyzed studies, 10 employed spoken reports to capture athletes’ subjective cognitive and psychological processes during decision-making. The oral reporting approach involves participants verbalizing their thoughts, emotions, and decisions while performing a specific motor task or encountering a particular situation. In three studies conducted by Kittel and colleagues, referees were instructed to sit in swivel chairs for safety, aiming to minimize the risk of falling while observing virtual scenarios. Subsequently, participants were prompted to verbalize their decisions upon the presentation of the ‘Make a decision’ prompt on the screen, and the research team immediately recorded their responses (Kittel et al., 2019, 2020, 2021).

In motor decision-making research, the behavioral response approach primarily involves observing and recording participants’ actual behavior during motor tasks. This approach emphasizes individual actions, reaction time, motor skills, and the decision-making process in specific motor situations. Scientifically monitoring and identifying subjects’ behavioral actions are crucial aspects of the study. Some studies have utilized motion capture technology to record subjects’ decision-making actions. For instance, in a virtual basketball decision-making test, Tsai et al. utilized IMU sensors, with athletes selecting action decisions based on provided defensive scenarios. The IMU sensor data were recorded by an inertial motion capture system and recognized in real-time by a model (Tsai et al., 2021). Similarly, Gray installed a position tracker sensor on the baseball bat in their study to record its position. He then compared the ball’s position in the virtual environment with the recorded bat position in real-time to detect collisions (Gray, 2017).

In addition to the previously mentioned motor decision-making task interactions, some studies have employed a keystroke selection method. This method primarily evaluates participants’ speed and accuracy in decision-making within a specific context. Participants use a keyboard, joystick, or another input device to press relevant keys in response to a specific stimulus or task. For example, Van Maarseveen et al. conducted a virtual decision-making test where, after a decision-making video clip, a reaction slide displayed buttons for four possible options: shooting, dribbling, passing to the left teammate, and passing to the right teammate. Subjects were required to choose the optimal option for the ball carrier and press the corresponding button (Van Maarseveen et al., 2018).

## 4 Discussion

### 4.1 Reasons for using VR to assess and train sports decision-making

Previous research predominantly utilized traditional screen videos for assessing and training athletes’ decision-making abilities, but their ecological validity has been questioned (Lorains et al., 2013; Jin et al., 2023). The Modified Perceptual Training Framework (Hadlow et al., 2018) emphasizes that the transfer of perceptual-cognitive training relies on the similarity between the training model and the target skill. While efforts can be made to enhance correspondence between video simulations and real-life stimuli, an inherent difference in stimulus-visual correspondence remains. This distinction may contribute to the reduced immersive experience of video simulations on TV or computer screens (Pagé et al., 2019). Transferring improved decision-making abilities from traditional on-screen video training to real matches may present challenges. This is because visual-perceptual-motor responses elicited by traditional on-screen video may not entirely replicate those in real-game situations, considering the three-dimensionality of human vision (Craig, 2013). Traditional on-screen video training has limitations, often presented from a third-person viewpoint, disrupting the relationship between the environment and the observer during movement, leading to decreased stimulus correspondence. To address this limitation, consider using self-centered videos that adjust to the viewer’s head movements (Panchuk et al., 2018). Starkes and Lindley (1994) asserted that perceptual-cognitive training using traditional videos failed to translate into improved decision-making in actual game contexts, primarily because during training, players viewed game scenarios from a spectator’s perspective, not as participants in an actual basketball game (Lorains et al., 2013).

Virtual reality, a computer-simulated representation of real or imagined environments, enables users to interact with virtual situations, enhancing visual correspondence in video simulations and improving perceptual-cognitive skills (Craig, 2013). In recent years, virtual reality environments with high ecological validity and psychological fidelity have become the preferred experimental tool for researchers investigating decision-making. The study suggests that an immersive VR video-based approach is more effective in assessing and training athletes’ motor decision-making abilities than a traditional screen-based video approach. To optimize the effectiveness of decision-making training, tasks should be designed to accurately simulate real-world environments.

Sports environments in virtual reality, created through VR technology, offer a more accurate simulation of real sports compared to traditional video, leading to increased immersion, concentration, and engagement (Pagé et al., 2019). This is due to the advanced technology integrated into VR, which enhances the sense of realism. The study showed that training sports decision-making in virtual reality significantly outperformed traditional video training in improving athletes’ decision-making speed. However, there was no significant difference between the two methods in terms of improving decision-making quality. This may be attributed to the low complexity of the motor decision-making task scenarios used and the potential repetition of specific scenarios encountered in previous assessments during the post-training test (Hohmann et al., 2016). Additionally, the feedback provided to athletes is limited to the decision-making options presented in the video. Providing high-level athletes with feedback on additional potential solutions to the task could enhance the quality of their decision-making more effectively.

### 4.2 Choosing the appropriate presentation method for virtual environments

The studies reviewed demonstrate that the presentation methods for constructing virtual environments for sports decision-making tasks primarily involve 360-degree VR and Animated VR, delivered through HMDs or CAVE projection systems. Despite the application of these methods in various studies, significant differences exist between them. To improve the efficacy of future research, this section focuses on delineating these differences and provides guidance for researchers to select the most suitable presentation method for virtual reality tasks, based on the specific requirements of their studies.

In recent studies, HMD virtual reality devices have been used in 15 investigations, whereas CAVE projection systems have been chosen in only 5 studies. A primary reason for this difference is cost, as CAVE projection systems require a significant laboratory space, which is often considered a valuable resource (Havig et al., 2011). CAVE systems need six equally sized projection walls, and the cost of high-resolution projectors is substantial. Additionally, participants must wear passively polarized 3D glasses to achieve a three-dimensional effect. In contrast, the HMD has a smaller footprint than the CAVE, occupying only the wearer’s area. Furthermore, it offers superior resolution and a wider field of view, tracking the user’s head movements and resulting in an enhanced visual perspective (Mallaro et al., 2017). While both HMDs and CAVE systems provide immersive experiences, the smaller size, portability, and lower cost significantly contribute to the current prevalence of HMDs (Mallaro et al., 2017).

Regarding the presentation methods of 360-degree VR and Animated VR, research indicates that there is no significant difference in their effectiveness for assessing and training sports decision-making (Tsai et al., 2021). During the viewing of 360-degree panoramic videos on an HMD, users can adjust the viewing angle by turning their heads, allowing for deeper immersion in the specific panorama. Both 360-degree panoramic videos and computer-simulated 3D virtual content provide athletes with a realistic training experience, each with its own set of advantages and disadvantages. In terms of the visual experience, 360-degree panoramic videos capture authentic scenes, enabling athletes to immerse themselves more deeply in real training scenarios compared to computer-generated simulations. However, as video content must be pre-recorded, athletes cannot modify their movements once the camera is fixed in a first-person perspective. On the other hand, in a computer-simulated virtual space, athletes have the freedom to move within the virtual scenario. Regarding feasibility, creating ideal panoramic training scenarios through 360-degree video shooting is a time-intensive process. In contrast, generating diverse and controllable training scenarios through computer simulation is considerably more convenient. The study found no significant differences in knowledge acquisition, decision time, and the level of presence between virtual environments created using 360-degree panoramic videos and computer simulations (Tsai et al., 2021).

**Figure 2:**
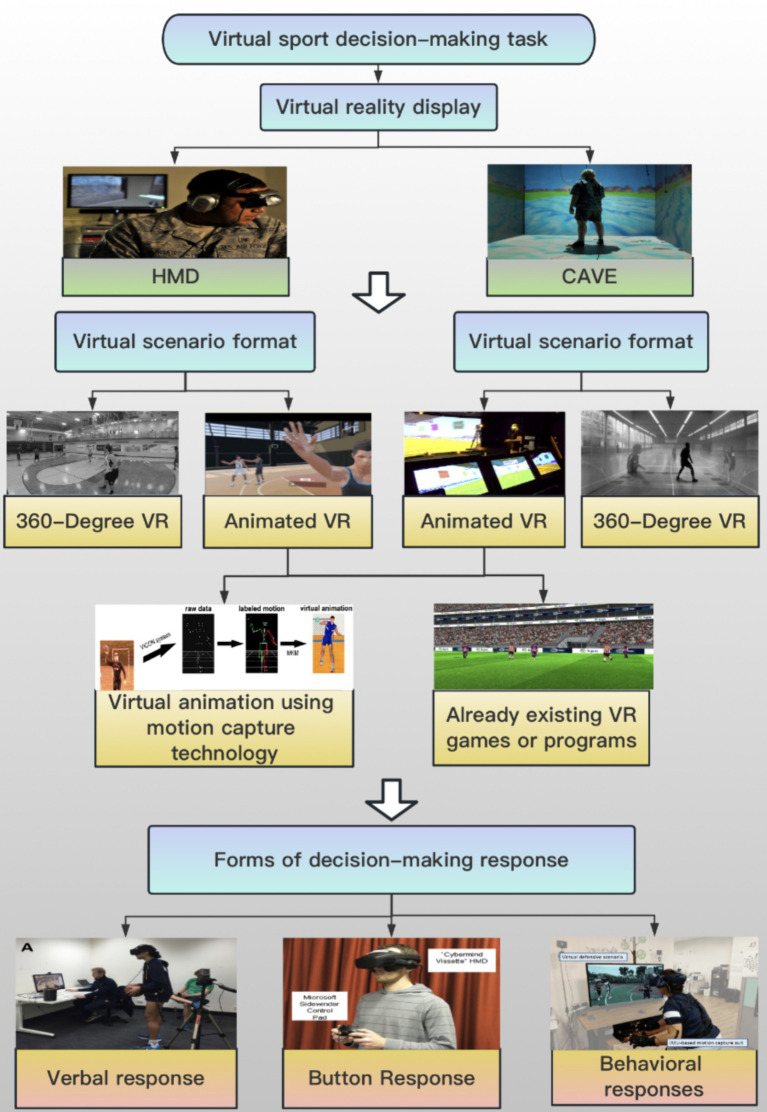
Flowchart for creating a virtual sports decision-making scenario (Vignais et al., 2015; Panchuk et al., 2018; Pagé et al., 2019; Magnaguagno and Hossner, 2020).

Both 360-degree VR and Animated VR have inherent limitations. 360-degree VR does not permit user interaction with the environment, creating a disconnect between perception and action that may hinder skill development. Animated VR, on the other hand, faces challenges in accurately simulating virtual objects to behave as they would in the real world, potentially affecting the realism of the perceptual-action coupling (Le Noury et al., 2021). Tsai proposed in the study that, in the future, researchers may consider using computer-simulated content to develop more comprehensive virtual training scenarios, considering the substantial time and labor required for creating efficient and detailed 360-degree decision-making training videos (Tsai et al., 2021).Virtual scenarios can be simulated in a computer-generated environment using a motion capture system to replicate a character in a specific sporting situation (Vignais et al., 2015). For example, Vignais et al. (2015) captured kinematic data of a throwing movement at 100 Hz using the VICON MX40 motion capture system, which includes 12 infrared cameras. Kinematic data from the throwing motion were labeled and transferred to the MKM (Manageable Kinematic Motions) animation engine (Multon et al., 2008). In the VE experiment, a synthetic humanoid was animated in a handball stadium scaled with real dimensions (see Figure 2; Vignais et al., 2015). Furthermore, existing VR games related to sports can be utilized to recreate sport scenarios (Marshall et al., 2023). In Vignais’ study, The Rezzil Player 22 (Rezzil Europe, Manchester, United Kingdom) application was used to provide VR football heading training. This application consists of 60 heading training drills with high scores for consistency and accuracy allowing progress to further drills (Vignais et al., 2015). In summary, Animated VR allows athletes to interact with the sports scene, benefiting the coupling between perception and action. However, creating Animated VR sports scenes requires a certain level of computer expertise, posing a challenge for future sports researchers. Therefore, researchers need to select the most suitable presentation method for virtual sports scenes based on the specific sport, objectives, technical expertise, available equipment, and other relevant factors.

### 4.3 Optimal interaction for VR sports decision-making tasks

In team sports, athletes often face unpredictable and intricate situations that demand rapid and efficient decision-making. This skill is crucial for enhancing athletes’ decision-making capabilities. The research presented in this text sheds light on how athletes engage with virtual decision-making tasks, including verbal reports, keystroke selections, and behavioral responses.

The Modified Perceptual Training Framework (MPTF) underscores the significant importance of an athlete’s response to a task stimulus in improving perceptual function during training. Correspondence in responses is vital, indicating the alignment between required responses in perceptual training and the level of response in real-world activities. Hadlow et al. suggest that athletes engaged in physical actions establish a stronger connection between perception and action, resulting in superior perceptual-cognitive performance compared to those providing verbal or written responses (Hadlow et al., 2018). Wirth et al. observed that action-behavior responses outperformed verbal interactions in their study. Additionally, activating muscle tension during the training of specific mental and cognitive skills has a positive impact on athletes’ training outcomes (Wirth et al., 2018).

The validity of virtual decision-making scenarios is lower compared to real sports scenarios, especially when athletes only judge perception without taking corresponding actions. Comparing results between perception tasks and perception-action tasks did not produce identical findings. Thus, while the authenticity of decision-making scenarios is important, the authenticity of behavior may be even more crucial for athletes (Craig, 2013). Furthermore, precise monitoring, capturing, and recognizing athlete actions are essential for employing action-behavioral responses. Among the examined studies, only six used a motion-behavior response approach, all using motion capture devices or position trackers with various technologies. However, it’s important to note that the accuracy of a motion capture system can be affected by several factors, including the performance of the computer server and the quantity and quality of cameras used. Adjusting the number of markers and sampling frequency can significantly improve the accuracy of the motion capture system. Recent research has demonstrated that optically-based motion capture systems offer high accuracy and minimal response time, crucial for providing real-time feedback to the user.

### 4.4 Exploring the internal characteristics of athletes’ decision-making using VR

Athletes heavily rely on visual cues to make decisions in sports situations. Therefore, to effectively use VR in evaluating and enhancing athletes’ sports decision-making skills, understanding athletes’ visual characteristics is vital. Among the 20 studies reviewed, only four employed eye-tracking tools to assess athletes’ visual behaviors in virtual decision-making tasks. Researchers often focus on external aspects of athletes’ task responses, neglecting the internal aspects of their visual behavior. Analyzing gaze behavior enhances our understanding of the relationship between various perceptual-cognitive skill assessments and the internal processes that impact task performance (Broadbent et al., 2015). Therefore, recording athletes’ visual behavior information when using VR to assess their decision-making abilities is extremely important.

In previous studies, VR combined with eye-tracking technology has been used to analyze athletes’ eye movements during decision-making tasks, aiming to identify and evaluate athletes’ skill levels. Hosp et al. proposed a model for identifying the expertise of soccer goalkeepers in game situations, employing a machine learning algorithm based solely on eye movements. Athletes’ visual patterns contain highly informative features, enabling the classification of soccer goalkeepers into three expertise levels: elite, professional, and novice players, with an accuracy of recognition reaching 78.2%. This study underscores the importance of utilizing eye tracking and machine learning to explore non-sensory expertise, laying the groundwork for sensory-cognitive assessment and training systems (Hosp et al., 2021). Furthermore, recording athletes’ gaze behavior during perceptual-cognitive skill tests can enhance our understanding of whether their task performance correlates with internal processes. The study by Van Maarseveen et al. (2018) demonstrated that it is challenging to predict athletes’ on-field performance based solely on perceptual-cognitive test scores, as these recall patterns are influenced by internal processes distinct from expectation and decision-making.

Due to the strong link between eye movements and cognition, eye tracking has garnered increasing attention across various experiments (Clay et al., 2019). With the ongoing evolution of measurement devices and their integration into VR systems, an expanding array of devices now incorporate eye trackers within HMDs to create virtual eye trackers (Hosp et al., 2021). This technology enables precise measurement of user gaze behavior while presenting highly realistic stimulus tasks. Eye-tracking instruments provide a robust foundation for studying perceptual processes with high temporal and spatial resolution, facilitating the collection of extensive and intricate data (Castner et al., 2020). Utilizing machine learning techniques to analyze eye-tracking data gathered from observing virtual scenes in HMDs is essential for developing virtual reality decision training systems. In specific eye-tracking tasks such as scene perception, visual search, and task-driven actions, it’s possible to analyze the user’s eye-movement map in greater detail based on the task methodology (Pastel et al., 2023). The integration of eye-tracking and VR allows for the calculation of the subject’s gaze point in a virtual 3D space and observation of the subject’s gaze point position during task movement. Visual search behavior is integral to the decision-making process (Ugwitz et al., 2022). Pagé et al. (2019) suggest that under VR conditions, athletes may enhance their ability to search for and acquire visual information. The scene displayed by the head-mounted display (HMD) alters with head movements, offering higher visual correspondence. This increase in visual correspondence boosts athletes’ motivation to search for information and actively seek information-rich areas, subtly refining their methods of acquiring visual information. The benefits of decision-making training derived from this approach are not confined to specific game scenarios but extend to all situations on the field.

The fusion of eye-tracking technology with virtual reality offers numerous advantages, including more natural and realistic stimuli, controlled environments, and data collection, opening avenues for exploring research questions in innovative ways. When evaluating athletes’ motor decision-making abilities using a virtual decision-making task assessment system, the virtual eye-tracker’s capability to analyze the information characteristics of the athlete’s internal processes during this decision-making process can provide a comprehensive understanding of their behavioral choices. Virtual eye-tracking instruments hold significant research potential for investigating the interconnectedness of human perception, cognition, and behavior (Ugwitz et al., 2022).

## 5 Conclusion

This systematic review compiles findings from 20 studies investigating the role of VR technology in sports decision-making within team sports. The aim is to offer a comprehensive understanding of the effectiveness, limitations, and future potential of this technology in the field. The results indicate that VR technology serves as a valuable tool for evaluating and improving athletes’ motor decision-making skills in team sports. When presenting virtual scenarios, HMDs demonstrate greater efficiency compared to CAVE systems, due to their compactness, portability, and cost-effectiveness. Given the significant time and resources needed to create detailed 360-degree panoramic videos, future research could explore using computer-generated content to develop more comprehensive virtual motion scenarios tailored to specific mission requirements. Regarding virtual task interaction in sports decision-making, it is beneficial to allow athletes to make judgments in decision-making tasks while simultaneously executing corresponding actions, as this can enhance the effectiveness of skill transfer. In studies focusing on perceptual-cognitive tasks, it is vital to consider athletes’ visual behavioral characteristics in motor task processing. Visual perception should be combined with other sensory inputs to investigate motor decision-making thoroughly. The integration of eye-tracking and VR technology offers insights into the intrinsic link between cognition and action in athletes.

## Data availability statement

The original contributions presented in the study are included in the article/supplementary material, further inquiries can be directed to the corresponding author.

## Author contributions

YJ: Conceptualization, Data curation, Formal analysis, Funding acquisition, Investigation, Methodology, Project administration, Resources, Software, Supervision, Validation, Visualization, Writing – original draft, Writing – review & editing. XZ: Conceptualization, Data curation, Investigation, Supervision, Validation, Writing – review & editing. JY: Project administration, Supervision, Validation, Writing – review & editing. QF: Conceptualization, Funding acquisition, Investigation, Project administration, Resources, Supervision, Validation, Visualization, Writing – review & editing.
